# Study protocol: mycophenolate mofetil as maintenance therapy after rituximab treatment for childhood-onset, complicated, frequently-relapsing nephrotic syndrome or steroid-dependent nephrotic syndrome: a multicenter double-blind, randomized, placebo-controlled trial (JSKDC07)

**DOI:** 10.1186/s12882-018-1099-7

**Published:** 2018-11-01

**Authors:** Tomoko Horinouchi, Mayumi Sako, Koichi Nakanishi, Kenji Ishikura, Shuichi Ito, Hidefumi Nakamura, Mari Saito Oba, Kandai Nozu, Kazumoto Iijima

**Affiliations:** 1 Department of Pediatrics, Kobe University Graduate School of Medicine, 5-1 Kusunoki-cho 7 chome, Chuo-ku, Kobe, 650-0017 Japan; 2 Division for Clinical Trials, Department of Clinical Research Promotion, Clinical Research Center, National Center for Child Health and Development, Tokyo, Japan; 3 Department of Child Health and Welfare (Pediatrics), Graduate School of Medicine, University of the Ryukyus, Okinawa, Japan; 4 Division of Nephrology and Rheumatology, National Center for Child Health and Development, Tokyo, Japan; 5 Department of Pediatrics, Graduate School of Medicine, Yokohama City University, Yokohama, Japan; 6 Clinical Research Center, National Center for Child Health and Development, Tokyo, Japan; 7 Department of Medical Statistics, Toho University, Tokyo, Japan

**Keywords:** Mycophenolate mofetil, rituximab., frequently-relapsing nephrotic syndrome., steroid-dependent nephrotic syndrome., steroid-sensitive nephrotic syndrome., multicenter, double-blind, randomized, placebo-controlled trial.

## Abstract

**Background:**

Idiopathic nephrotic syndrome (INS) is the most common chronic glomerular disease in children. Approximately 80–90% of patients with childhood INS have steroid-sensitive nephrotic syndrome (SSNS), and can obtain remission with steroid therapy, while the remainder have steroid-resistant nephrotic syndrome (SRNS). Furthermore, approximately 50% of children with SSNS develop frequently-relapsing nephrotic syndrome (FRNS) or steroid-dependent nephrotic syndrome (SDNS). Children with FRNS/SDNS are usually treated with immunosuppressive agents such as cyclosporine, cyclophosphamide, or mizoribine in Japan. However, 10–20% of children receiving immunosuppressive agents still show frequent relapse and/or steroid dependence during or after treatment, which is defined as complicated FRNS/SDNS. Furthermore, 30% of SRNS patients who obtain remission after additional treatments such as cyclosporine also turn out to be complicated FRNS/SDNS. For such complicated FRNS/SDNS patients, rituximab (RTX) is currently used; however, recurrence after RTX treatment also remains an open issue. Because long-term use of existing immunosuppressive drugs has limitations, development of a novel treatment for maintenance therapy after RTX is desirable. Mycophenolate mofetil (MMF) is an immunosuppressive drug with fewer side effects than cyclosporine or cyclophosphamide. Importantly, recent studies have reported the efficacy of MMF in children with nephrotic syndrome.

**Methods:**

We conduct a multicenter, double-blind, randomized, placebo-controlled trial to evaluate the efficacy and safety of MMF after RTX therapy in children with complicated FRNS/SDNS. Patients are allocated to either RTX plus MMF treatment group, or RTX plus placebo treatment group. For the former group, MMF is administered at a dose of 1000–1200 mg/m^2^/day (maximum 2 g/day) twice daily for 17 months after RTX treatment. The primary endpoint is time-to-treatment failure (development of frequent relapses, steroid dependence or steroid resistance).

**Discussion:**

The results will provide important data on the use of MMF as maintenance therapy after RTX to prevent complicated FRNS/SDNS patients from declining into treatment failure. In future, MMF in conjunction with RTX treatment may permit increased duration of remission in ‘complicated’ FRNS/SDNS cases.

**Trial registration:**

This trial was prospectively registered to UMIN Clinical Trials Registry on June 23, 2014 (UMIN Trial ID: UMIN000014347).

## Background

Childhood-onset idiopathic nephrotic syndrome (INS) is the most common glomerular disease which occurs in more than 2 cases/100,000 children [1]. Notably, in Japan, the estimated incidence of INS is 6.49 cases/100,000 children annually [2]. Minimal change nephrotic syndrome is the most common form of the disorder, for which steroid therapy is effective for most patients [3]. Eighty to 90 % of patients achieve remission with the administration of steroids (steroid-sensitive nephrotic syndrome; SSNS) while 10–20% of patients suffer from steroid-resistant nephrotic syndrome (SRNS) which does not achieve remission with the administration of steroids [4].

Those who respond well rarely progress to end stage renal disease, but up to 50% of SSNS cases develop frequently-relapsing nephrotic syndrome (FRNS) [5]. FRNS is defined as at least four relapses per year or at least two within 6 months of the initial presentation (Table 2) [6]. A total of 50–60% of children with FRNS develop two consecutive relapses during tapering or within 14 days of stopping steroid therapy, this is termed steroid-dependent nephrotic syndrome (SDNS) (Table 2) [3, 6]. Treatment with immunosuppressive drugs is carried out to avoid the steroid-specific adverse event because each relapse requires a large dose of steroids. The Kidney Disease: Improving Global Outcomes Clinical Practice Guideline for Glomerulonephritis recommends alkylating agents, such as cyclophosphamide or chlorambucil, levamisole, calcineurin inhibitors, including cyclosporine or tacrolimus, and mycophenolate mofetil (MMF) as corticosteroid-sparing agents for children with FRNS/SDNS [7]. The clinical practice guidelines for pediatric idiopathic nephrotic syndrome (2013) recommend cyclosporine, cyclophosphamide, and mizoribine as immunosuppressive drugs for FRNS/SDNS [6]. Most children with FRNS/SDNS are effectively treated with these recommended immunosuppressant drugs; however, at least 10–20% of children receiving immunosuppressive agents still show frequent relapses or steroid dependence after treatment (complicated FRNS/SDNS). Additionally, some patients with SRNS develop steroid-sensitive frequent relapses or steroid dependence after achievement of complete remission by immunosuppressive therapies including calcineurin inhibitors (complicated FRNS/SDNS). A 5-year follow-up study of cyclosporine treatment in children with SRNS showed that 7 of 31 (23%) patients developed frequent relapses under immunosuppressive therapy after achievement of complete remission [8]. Meanwhile, the total dosage of cyclophosphamide is restricted due to gonadal toxicity and late-onset carcinogenicity, and we cannot use cyclosporine exclusively because cyclosporine can cause chronic nephrotoxicity as a side effect. However, discontinuation of cyclosporine often results in frequent relapses again [9, 10].

In that context, there have been many reports that rituximab (RTX), a monoclonal antibody directed against the CD20 differentiation antigen expressed on the surface of B cells, is effective and safe in children with complicated FRNS/SDNS [11–13]. Recently, RTX has been used for complicated FRNS/SDNS, although some cases tend to relapse after the recovery of B cell counts [14–16]. In addition, the safety of long-term B cell suppression caused by repeated administration of RTX in children whose immune system is developing is unknown. Therefore, a new maintenance therapy to prevent the relapse after RTX treatment is urgently needed.

Mycophenolate mofetil (MMF) is an immunosuppressant which selectively blocks de novo purine synthesis, a pathway crucial for both B and T lymphocytes, and has been used for various autoimmune diseases and as immunosuppressive therapy after organ transplantation [17–20]. In addition, it is reported that MMF is effective in childhood-onset nephrotic syndrome [21–30]. Our group has conducted a pilot study and reported that maintenance therapy with MMF after a single dose of RTX in complicated SDNS significantly prolonged the relapse-free period compared with RTX monotherapy [31]. Thus, MMF is a promising drug for maintenance therapy after RTX, however a prospective randomized clinical trial is still needed. [32] Therefore, we conduct a multicenter, double-blind, randomized, placebo-controlled trial to evaluate the efficacy and safety of MMF after RTX therapy in children with complicated FRNS/SDNS.

## Methods/design

**Fig. 1:**
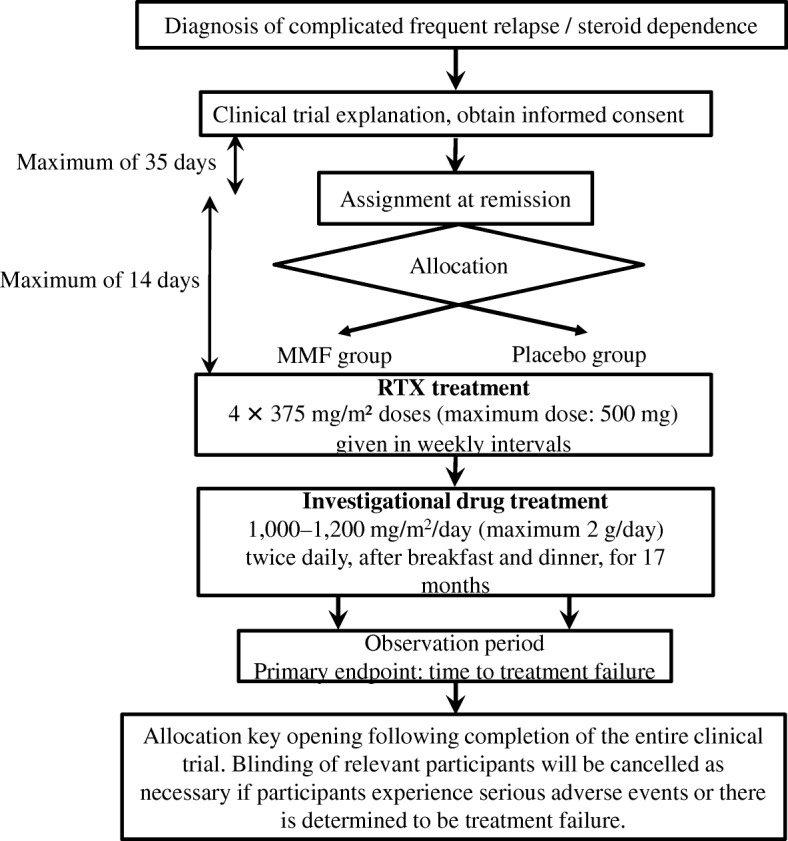
Flow diagram of the clinical trial set-up. This trial is a multicenter, double-blind, randomized, placebo-controlled trial. After obtaining informed consent, registration and allocation is conducted. After rituximab treatment, mycophenolate mofetil or placebo is administered and the treatment key is opened following completion of the entire clinical trial

A flow chart of the study design is shown in Fig. 1.

### Aim

The aim of this trial is to investigate whether RTX plus MMF combination therapy is superior to RTX alone for the maintenance of remission after RTX treatment in children with complicated FRNS/SDNS.

### Study design and patients

We conduct a multicenter, double-blind, randomized, placebo-controlled trial to assess the efficacy and safety of MMF after RTX therapy in children with complicated FRNS/SDNS (Fig. 1, Table 2 [6]). In total, 80 patients from 27 institutions in Japan will be enrolled in this study. We will diagnose NS and remission and relapse according to the International Study of Kidney Disease in Children (ISKDC) [6, 33]. Patients who are aged between 1 and 18 years old at the time of onset of idiopathic nephrotic syndrome and are aged 2 years or above at the time of registration are eligible if they fall into the complicated FRNS/SDNS category.

Inclusion and exclusion criteria are as follows:

Inclusion criteria:

1. Diagnosed as INS according to the ISKDC criteria.
2. The initial onset of INS is at between 1 and 18 years of age, and the patient is 2 years of age or older at assignment.
3. Patients meeting one of the following criteria:
  1. Diagnosed with frequent relapse or steroid dependence and once again diagnosed with frequent relapse or steroid dependence after completion of immunosuppressive drug therapy (cyclosporine, cyclophosphamide, or mizoribine, etc.).
  2. Diagnosed with frequent relapse or steroid dependence and once again diagnosed with frequent relapse or steroid dependence during immunosuppressive drug therapy (cyclosporine, cyclophosphamide, or mizoribine, etc.).
  3. Diagnosed with steroid resistance following the onset of INS and diagnosed with frequent relapse or steroid dependence during or after the completion of immunosuppressive drug therapy (cyclosporine alone or combination of cyclosporine and methylprednisolone, etc.).
4. Patients with records of the nearest preceding 3 relapses.
5. Patients in whom steroid sensitivity is observed during treatment of relapse immediately prior to assignment.
6. Patients in whom ≥5 CD20-positive cells/μL are observed in the peripheral blood.
7. Patients who can be hospitalized overnight on the first day of rituximab administration.
8. Written informed consent.

Exclusion criteria:

1. Patients who have been diagnosed with nephritic-NS, such as IgA nephropathy, prior to assignment or in whom secondary NS is suspected.
2. Patients who have used a monoclonal antibody other than rituximab.
3. Patients meeting one of the following infection criteria:
  1. Presence or history of severe infections within 6 months prior to assignment.
  2. Presence or history of opportunistic infections within 6 months prior to assignment.
  3. Presence of active tuberculosis.
  4. Patients with a history of tuberculosis or in whom tuberculosis is suspected.
  5. Presence or history of active hepatitis B or hepatitis C or hepatitis B virus carrier.
  6. Presence of human immunodeficiency virus (HIV) infection.
4. Presence or history of angina pectoris, cardiac failure, myocardial infarction, or serious arrhythmia (findings observed under Grade 4 of the Common Terminology Criteria for Adverse Events (CTCAE)).
5. Presence or history of autoimmune diseases or vascular purpura.
6. Presence or history of malignant tumor.
7. History of organ transplantation.
8. History of drug allergies to methylprednisolone, acetaminophen, or d-chlorpheniramine maleate.
9. Uncontrollable hypertension.
10. Deteriorated kidney function, e.g. estimated glomerular filtration rate (GFR) < 60 mL/min/1.73 m^2^.
11. Having received a live vaccine within 4 weeks prior to enrollment.
12. Patients showing one of the following abnormal clinical laboratory values:
  1. Leukocytes < 3000/μL.
  2. Neutrophils < 1500/μL.
  3. Platelets < 50,000/μL.
  4. Alanine aminotransferase (ALT) > 2.5× upper limit of normal value.
  5. Aspartate aminotransferase (AST) > 2.5× upper limit of normal value.
  6. Positive for hepatitis B surface (HBs) antigen, HBs antibody, hepatitis B core (HBc) antibody, or HCV antibody.
  7. Positive for HIV antibody.
13. Patients who do not agree with contraception during the study period.
14. Women during pregnancy or breast-feeding.
15. Judged inappropriate for this study by the treating or study physicians.

### Randomization

Patients are randomly assigned to either RTX plus MMF, or RTX plus placebo group at an approximate ratio of 1:1 using the following allocation adjustment factors; medical institution, age, treatment history (presence or absence of immunosuppressive drug administration at the relapse immediately prior to enrollment, presence or absence of steroid administration at the relapse immediately prior to enrollment), interval between the last 3 relapses, presence or absence of history of SRNS.

Patients, their guardians, treating physicians, and individuals assessing outcomes and analyzing data are blinded to the patients’ assigned treatment. Apart from the data-monitoring committee, all treating physicians and other investigators remain blinded to the trial results until follow-ups are completed.

### Procedures

#### Observation period

The observation period is from the date the first dose of RTX is administered (day 1) to the date MMF administration is finished (day 505).

### Dosage regimen

**Fig. 2:**
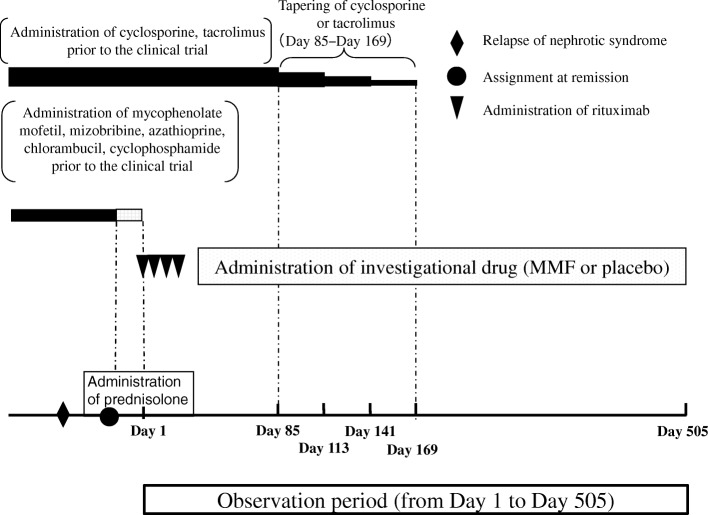
Dosage regimen. Rituximab (RTX) and investigational drug (mycophenolate mofetil (MMF) or placebo) are administered in this trial. The date the first dose of RTX is administered is defined as day 1 and the date MMF administration is finished will be day 505. Calcineurin inhibitors (cyclosporine, tacrolimus) administered prior to registration for this clinical trial are administered in combination with the trial regimen and dosage at the time of registration until day 85 (however the dosage may be changed based on monitoring) and the dosage will be sequentially reduced every 28 days from day 86 onwards and discontinued approximately on day 169. MMF, mizoribine, azathioprine, cyclophosphamide, chlorambucil administered prior to the registration of this clinical trial, are discontinued on day 1. Prednisolone will be administered for treatment of relapse immediately prior to registration or during the observation period

RTX and the investigational drug (MMF or placebo) are administered in this trial. The dosage regimen is shown in Fig. 2. For the RTX plus MMF combination group, RTX is administered as 4 × 375 mg/m^2^ doses (maximum dose: 500 mg) given at weekly intervals and MMF is additionally administered at a dose of 1000–1200 mg/m^2^/day (maximum 2 g/day) twice daily after breakfast and dinner, continued for 17 months after RTX treatment. For the RTX plus placebo group, RTX is administered as 4× 375 mg/m^2^ doses (maximum dose: 500 mg) given at weekly intervals and the placebo is administered in place of MMF.

The investigational drug can be begun at half the dose and increased to the defined dose in the absence of adverse reactions within 3 months. If patients cannot accept the full dose due to of adverse events, the doctors in attendance can decide the dose reduction. To prevent infusion reactions, patients receive a premedication with methylprednisolone, oral acetaminophen, oral d-chlorpheniramine maleate approximately 30 min prior to the administration of each dose of RTX [11].

### Prednisolone treatment for relapse at screening and during the study period

Participants receiving prednisolone for relapse at screening continue receiving the drug, taking 60 mg/m^2^ orally three times a day (maximum of 80 mg per day or 60 mg per day, depending on the institution policy) for 4 weeks. Participants with relapse not receiving prednisolone at screening receive the same dose until 3 days after complete remission is achieved. After 4 weeks (in patients who received prednisolone at screening) or from 3 days after complete remission (in patients who did not receive prednisolone at screening), patients take 60 mg/m^2^ prednisolone in the morning on alternate days (maximum 80 mg per day or 60 mg per day) for 2 weeks, then 30 mg/m^2^ on alternate days (maximum 40 mg per day or 30 mg per day) for 2 weeks, and then 15 mg/m^2^ on alternate days (maximum 20 mg per day or 15 mg per day) for 2 weeks. When patients have relapses during the study period, they receive 60 mg/m^2^ oral prednisolone three times a day (maximum 60 mg per day) until 3 days after complete remission is obtained, then take 60 mg/m^2^ prednisolone in the morning on alternate days (maximum 60 mg per day) for 2 weeks, then 30 mg/m^2^ on alternate days (maximum 30 mg per day) for 2 weeks, and then 15 mg/m^2^ on alternate days (maximum 15 mg per day) for 2 weeks.

### Concomitant dugs and combination therapy

In the event patients are receiving a calcineurin inhibitor (cyclosporine or tacrolimus) at screening, tapering of the drug begins at day 86, with discontinuation by day 169; the dosage will be sequentially reduced every 28 days from day 86 onwards and discontinued on approximately day 169 (Fig. 2). If patients were taking any other immunosuppressive agents (MMF, mizoribine, azathioprine, cyclophosphamide or chlorambucil), these drugs are discontinued by the beginning of RTX administration (day 1) (Fig. 2).

Trimethoprim-sulfamethoxazole is administered from the beginning of RTX treatment (day 1) until the date on which peripheral blood B cell recovery (≥ 5 cells/μL) is confirmed for the prevention of *Pneumocystis jirovecii* infection.

Combination therapy with the following drugs and treatment are prohibited during the clinical trial period.

1. Commercially available rituximab.
2. Immunosuppressive drugs or alkylating agents with an immunosuppressive effect except in the following cases.
  1. In the case “cyclosporine, tacrolimus, cyclophosphamide, mizoribine, MMF or chlorambucil” continues to be used from prior to the start of the clinical trial.
  2. In the case treatment failure is determined.

## Live vaccines

### The discontinuation of investigational drug administration

Investigators are to discontinue the administration of investigational drugs to participants to whom any of the following circumstances apply:

1. If treatment failure (FRNS, SDNS, or SRNS) is observed during the observation period.
2. If prohibited drug 1.2. (see above) is used as a treatment for nephrotic syndrome.
3. If the participant or legal representative requests discontinuation of the administration of the investigational drug.
4. If the investigators determine the continuation of investigational drug administration to be difficult for any other reason such as the occurrence of adverse events.
5. If the participant becomes pregnant.

### Visit schedule

During the clinical trial period, investigators carry out observations, examinations, and surveys in accordance with the prescribed schedule. The visit schedule is shown in Table 1. Study visits occur every week during the RTX administration period, every 1 month during the first 6 month of the investigational drug administration period, and every 2 month, thereafter. Urine samples and blood samples are taken every visit.

**Table 1:** Clinical trial schedule

	Screening period	Observation period				Observation period														Follow-up period
		(Rituximab administration period)				(Investigational drug administration period)														
Day	Within 35 days	1	8	15	22	29	57	85	113	141	169	225	281	337	393	449	505	Relapse	Investigation drug discontinuation	36/48 month Clinical trial discontinuation
Visit		1	2	3	4	5	6	7	8	9	10	11	12	13	14	15	16			
Obtaining informed consent	○																			
Medical examination	○	○	○	○	○	○	○	○	○	○	○	○	○	○	○	○	○	○	○	
Investigation drug administration		○	○	○	○	○	○	○	○	○	○	○	○	○	○	○	○	○	○	
Background survey	○																			
Concomitant drug survey	○	○	○	○	○	○	○	○	○	○	○	○	○	○	○	○	○	○	○	
Height/weight	○	○	○	○	○	○	○	○	○	○	○	○	○	○	○	○	○	○	△	○
Blood pressure	○	○	○	○	○	○	○	○	○	○	○	○	○	○	○	○	○	○	△	○
Pulse, body temperature	○	○	○	○	○	○					○			○			○		△	
Pregnancy test	○																			
HIV, HCV, HBV^a^	○																			
Electrocardiogram	○																○		△	
Chest X-ray	○																○		△	
Relapse evaluation		○	○	○	○	○	○	○	○	○	○	○	○	○	○	○	○	○	○	○
Adverse event evaluation		○	○	○	○	○	○	○	○	○	○	○	○	○	○	○	○	○	○	○
After treatment																				○
Hematological examination	○	○	○	○	○	○	○	○	○	○	○	○	○	○	○	○	○	○	△	
Immunoglobulin examination		○					○		○		○			○			○		△	
Estimated glomerular filtration rate	○	○			○	○					○			○			○	○	○	
Urinalysis	○	○	○	○	○	○	○	○	○	○	○	○	○	○	○	○	○	○	○	○
Peripheral blood B cell count	○	○	○		○	○	○		○		○	○	○	○	○	○	○	○	○	□

△: Conduct if possible

□: Conduct until the peripheral blood B cell recovery (≥ 5/μL) is confirmed

^a^ *HIV* human immunodeficiency virus, *HCV* hepatitis C virus, *HBV* hepatitis B virus

### Allocation key opening

To maintain blinding, the “allocation codes” will be disclosed after the entire clinical trial is completed and all data and determination secured. However, if any of the following circumstances apply, the allocation code of the patient will be urgently disclosed.

1. The participant experiences a serious adverse event that leads to death or is life-threatening.
2. The participant experiences another serious adverse event and it is determined the information is essential in considering the relevant patient’s treatment.
3. There is determined to be treatment failure (FRNS, SDNS or SRNS).
4. The participant becomes pregnant and discontinues the administration of investigational drug.

### Outcomes

The primary endpoint is defined as the time-to-treatment failure (development of frequent relapses, steroid dependence or steroid resistance). Diagnosis of FRNS, SDNS, and SRNS is based on relapse dates according to the ISKDC (Table 2). The secondary end points are time to relapse, relapse rate, time to FRNS, time to SDNS, time to SRNS, total steroid dose, peripheral blood B cell depletion period and adverse events. Adverse events are recorded throughout the trial period and assessed using CTCAE.

**Table 2:** Definitions [6]

A. *Nephrotic syndrome*	The presence of a urine protein-to-creatinine ratio of 1.8 or above and serum albumin of 2.5 g/dL or below.
B. *Complicated nephrotic syndrome*	A patient that fulfills any of the following criteria is deemed to suffer from complicated nephrotic syndrome: (1) Diagnosed with frequent relapse or steroid dependence and once again diagnosed with frequent relapse or steroid dependence after completion of immunosuppressive drug therapy (cyclosporine, cyclophosphamide, mizoribine, etc.) (2) Diagnosed with frequent relapse or steroid dependence and once again diagnosed with frequent relapse or steroid dependence during immunosuppressive drug therapy (cyclosporine, cyclophosphamide, mizoribine, etc.) (3) Diagnosed with steroid resistance and diagnosed with frequent relapse or steroid dependence during or after the completion of immunosuppressive drug therapy (cyclosporine or combination of cyclosporine and methylprednisolone, etc.)
C. *Remission*	Negative protein on urine dipstick in the first morning urine for 3 consecutive days.
D. *Steroid sensitivity*	When the daily administration of prednisolone at 60 mg/m^2^/day leads to remission within 4 weeks.
E. *Relapse*	Protein 2+ or above detected by urine dipstick in the first morning urine for 3 consecutive days and prednisolone treatment is required.
F. *Frequent relapse*	Two or more relapses within 6 months after initial remission or 4 or more relapses within any 12-month period.
G. *Steroid dependence*	Two consecutive relapses during the reduction of steroid therapy or within 2 weeks of discontinuation of steroid therapy.
H. *Steroid resistance*	When the daily administration of prednisolone at 60 mg/m^2^/day does not lead to remission within 4 weeks.

### Statistical analyses

The primary aim of this study is to examine the superiority of RTX plus MMF combination therapy compared with RTX monotherapy in extending the duration to treatment failure. Based on a previous study, we hypothesize a 1-year event rate of 40% in the RTX treatment group and expect the RTX plus MMF treatment to decrease that to 20%. The planned sample size is 80 patients: 37 patients in each group will be needed to have 80% power for a log-rank test with a 5% significance level, under an assumption of proportional hazard rates, 3 years accrual, and one-and-a-half years follow-up. To allow for withdrawal of consent after participation in the study or loss to follow-up, we set the study size to 80 participants in total. The power calculation was performed using SAS version 9.3 (SAS Institute Inc., Cary, NC, USA). As a primary analysis, time-to-treatment failure is summarized using the Kaplan-Meier method and results compared using the log-rank test. The hazard ratios with 95% confidence intervals are estimated using Cox proportional hazard model. Secondary endpoints including time to relapse, time to FRNS, time to SDNS, time to SRNS, and B cell depletion period will be analyzed in the same manner as the primary endpoint. Model based analysis will be performed as needed. Relapse rate will be compared by permutation testing. Total steroid dose will be compared with the Wilcoxon test.

## Discussion

The purpose of this trial is to examine its safety and assess whether RTX plus MMF combination therapy is superior to RTX plus placebo for the maintenance of remission after RTX treatment in children with complicated FRNS/SDNS. Recently, RTX dramatically altered the treatment of complicated FRNS/SDNS [34]. Some patients can attain a ‘steroid-free period’ and/or ‘cyclosporine-free period’, while some cases tend to relapse after the recovery of B cell counts [14–16]. However, MMF has recently been focused on as a new treatment for childhood-onset nephrotic syndrome [21–30]. MMF is an immunosuppressive agent whose mechanism is similar to mizoribine in its inhibitory effect on the de novo pathway of nucleic acid synthesis [35]. In addition, we previously found that SDNS patients who do not relapse after RTX treatment were taking MMF [14]. Therefore, upon receiving the results of a pilot study, [31] we initiated a multicenter, double-blind, randomized, placebo-controlled trial to assess the efficacy and safety of MMF after RTX therapy in children with complicated FRNS/SDNS.

At present, there is a consensus that after RTX treatment, we do not use immunosuppressants until patients lapse into FRNS/SDNS again. Although the efficacy of MMF was shown in the pilot study, MMF is not an established treatment for maintenance therapy after RTX. Thus, it is reasonable to set the control group as immunosuppressant free. Simultaneously, we must pay careful attention so as not to restrict the chance for appropriate treatment in participants. To rescue the patients who fall into treatment failure, we are setting an urgent key open system. If a patient falls into FRNS, SDNS, or SRNS, the allocation code will be opened immediately and treatment conducted using immunosuppressants as soon as possible.

For complicated FRNS/SDNS patients, long-term treatment and multiple side effects are major issues. Current immunosuppressants such as calcineurin inhibitors, cyclophosphamide, mizoribine, and RTX have certainly helped patients, but existing treatments are not optimal. If we can demonstrate that MMF is safe and able to attain the long remission used for maintenance therapy after RTX, we will reduce the total dose of steroids, calcineurin inhibitors, and improve the quality of life in patients with complicated FRNS/SDNS. However, a limitation is that we cannot know the long-term prognosis of patients just by this trial, of which the observational period is just 18 months. In addition, MMF may not be curative like other existing treatments. Therefore, we must investigate the long-term prognosis of MMF in conjunction with RTX treatment while the discovery of treatments with a curative mechanism is also anticipated.

In conclusion, we conduct a multicenter, double-blind, randomized, placebo-controlled trial to evaluate the efficacy and safety of MMF after RTX therapy in children with complicated FRNS/SDNS. Results from this study may impact the management of pediatric complicated FRNS/SDNS patients. Improvement in quality of life will be accomplished by long-term remission, which should be of great benefit to both children with complicated FRNS/SDNS and their families.

## Abbreviations

CTCAE: Common Terminology Criteria for Adverse Events.
FRNS: Frequently-relapsing nephrotic syndrome
INS: Idiopathic nephrotic syndrome
ISKDC: International Study of Kidney Disease in Children
MMF: Mycophenolate mofetil
RTX: Rituximab
SDNS: Steroid-dependent nephrotic syndrome
SRNS: Steroid-resistant nephrotic syndrome
